# Long-term spaceflight and the cardiovascular system

**DOI:** 10.1093/pcmedi/pbaa022

**Published:** 2020-06-16

**Authors:** Nicholas A Vernice, Cem Meydan, Ebrahim Afshinnekoo, Christopher E Mason

**Affiliations:** The Bin Talal Bin Abdulaziz Alsaud Institute for Computational Biomedicine, Weill Cornell Medicine, 1305 York Avenue, New York, NY 10021, USA; The Bin Talal Bin Abdulaziz Alsaud Institute for Computational Biomedicine, Weill Cornell Medicine, 1305 York Avenue, New York, NY 10021, USA; Department of Physiology and Biophysics, Weill Cornell Medicine, 1300 York Avenue, New York, NY 10021, USA; The WordQuant Initiative for Quantitative Prediction, Weill Cornell Medicine, 1300 York Avenue, New York, NY 10021, USA; The Bin Talal Bin Abdulaziz Alsaud Institute for Computational Biomedicine, Weill Cornell Medicine, 1305 York Avenue, New York, NY 10021, USA; Department of Physiology and Biophysics, Weill Cornell Medicine, 1300 York Avenue, New York, NY 10021, USA; The WordQuant Initiative for Quantitative Prediction, Weill Cornell Medicine, 1300 York Avenue, New York, NY 10021, USA; The Bin Talal Bin Abdulaziz Alsaud Institute for Computational Biomedicine, Weill Cornell Medicine, 1305 York Avenue, New York, NY 10021, USA; Department of Physiology and Biophysics, Weill Cornell Medicine, 1300 York Avenue, New York, NY 10021, USA; The WordQuant Initiative for Quantitative Prediction, Weill Cornell Medicine, 1300 York Avenue, New York, NY 10021, USA; The Feil Family Brain and Mind Research Institute, Weill Cornell Medicine, 1300 York Avenue New York, NY 10021, USA

**Keywords:** microgravity, cardiovascular system, spaceflight, aerospace medicine

## Abstract

While early investigations into the physiological effects of spaceflight suggest the body's ability to reversibly adapt, the corresponding effects of long-term spaceflight (>6 months) are much less conclusive. Prolonged exposure to microgravity and radiation yields profound effects on the cardiovascular system, including a massive cephalad fluid translocation and altered arterial pressure, which attenuate blood pressure regulatory mechanisms and increase cardiac output. Also, central venous pressure decreases as a result of the loss of venous compression. The stimulation of baroreceptors by the cephalad shift results in an approximately 10%–15% reduction in plasma volume, with fluid translocating from the vascular lumen to the interstitium. Despite possible increases in cardiac workload, myocyte atrophy and notable, yet unexplained, alterations in hematocrit have been observed. Atrophy is postulated to result from shunting of protein synthesis from the endoplasmic reticulum to the mitochondria via mortalin-mediated action. While data are scarce regarding their causative agents, arrhythmias have been frequently reported, albeit sublethal, during both Russian and American expeditions, with QT interval prolongation observed in long, but not short duration, spaceflight. Exposure of the heart to the proton and heavy ion radiation of deep space has also been shown to result in coronary artery degeneration, aortic stiffness, carotid intima thickening via collagen-mediated action, accelerated atherosclerosis, and induction of a pro-inflammatory state. Upon return, long-term spaceflight frequently results in orthostatic intolerance and altered sympathetic responses, which can prove hazardous should any rapid mobilization or evacuation be required, and indicates that these cardiac risks should be especially monitored for future missions.

## Background

The pioneering spaceflights of Yuri Gagarin and John Glenn ushered in a new era of investigative potential for humanity, for both inside and outside the human body, as humans began to explore space. This excitement was accompanied by an avalanche of unknown, unpredictable risks and hazards to the human body during exploration of low-earth orbit (LEO), which continue to be explored until current day. Appropriately, protecting astronauts and cosmonauts quickly became central to the missions of both the American and Russian space programs of the early 1960s, with Europe, Japan, and China soon following suit. The general conclusion put forth by early space research suggested that the human body is capable of adaptation to spaceflight, albeit with many unknowns for long-term risks.^1,2^ Interestingly, with regards to long-term cardiovascular risk, NASA's Longitudinal Study of Astronaut Health, a retrospective and prospective cohort study from 1959 through 2010, concluded that short-duration spaceflight experience did not place NASA astronauts at a significantly increased risk for adverse cardiac events, including myocardial infarction, congestive heart failure, stroke, coronary artery disease, or coronary artery bypass surgery over the course of their lives with respect to that in their age, sex, and BMI-matched controls.^3^

Of great contemporary interest, however, is not the effect of simply being in space, but rather, the effect of living there. NASA's ambitions are now focused on launching a manned mission to the Moon (2024) and then Mars (2030s), upon which the ability of a human to successfully endure the extreme duress of that flight, its return, and re-acclimation to terrestrial conditions, is predicated. This is where negotiating the distinction between the effects of short-term spaceflight (<6 months), as experienced by Gagarin, Glenn, and the astronauts of the International Space Station (ISS), for example, and the effects of long-term, exploration-class spaceflight (>6 months, such as with Scott Kelly), to which any Mars-bound individual would undoubtedly be poised to endure, proves critical.^4^

## Physiological adaptation to spaceflight

Spaceflight of any duration places a wide variety of psychological and physical stressors on the body, including (but not limited to) sustained levels of ionizing radiation, circadian shifts, microgravity, diet restriction, sleep deprivation, reduced physical work, and confinement and isolation.^4^, ^5^ Many of the conditions have been demonstrated to yield profound effects on the physiology of the cardiovascular system.

Cardiovascular alterations from spaceflight begin immediately upon liberation from Earth's gravitational force, after which astronauts exhibit characteristically “puffy” faces, “stuffed” noses, and “chicken legs” as a result of microgravity-induced cephalad fluid shifts resulting from a decrease in intrathoracic pressure and movement of approximately 2 liters of fluid from the legs.^1,6^ In an upright position under the Earth's standard 1-*g* of gravity, arterial blood pressure is reduced above the heart and increased below the heart. However, in a microgravity environment, free from the Earth's gravitational gradient, the body experiences a uniform arterial pressure throughout, which reduces the physiological need for the body's blood pressure regulatory mechanisms and consequently decreases the cardiac workload.^7,8^ Within the first 24 hours of spaceflight, astronauts commonly experience a decrease in central venous pressure (CVP), a proxy measurement for cardiac filling pressure, increased cardiac chamber volumes, and increased atrial diameter caused by translocation of fluid to the head.^6,9^ The decrease in CVP is thought to result from the relief of the external compression usually applied by the internal viscera and musculature to the veins.^8^ Interestingly, decreases in CVP have been shown to occur simultaneously with increases in left ventricular end-diastolic volume.^9^ Although intuitively contradictory, later parabolic flights demonstrated a 1.3 mm Hg decrease in CVP for eight flight subjects during the weightlessness phase, while left atrial diameter increased by 3.6 mm and esophageal pressure saw a decrease of 5.6 mm Hg.^10,11^ Given that esophageal pressure is frequently used as proxy to measure intrathoracic pressure, the authors postulate that during weightlessness, transmural CVP, which is defined as the difference between CVP and intrathoracic pressure and indicates atrial distension pressure, increases, which might explain the increases in atrial diameter exhibited, despite decreases in CVP.^10,11^

As such, the massive fluid shift from the lower to the upper extremity resulting from the removal of the blood pressure gradient necessitates adaptation from the cardiovascular system, and the body's regulatory response to the massive fluid translocation is considerable. Perhaps most drastically, the distension of the heart and presence of redistributed fluid stimulates baroreceptors, resulting in inhibition of the renin-angiotensin-aldosterone system and an increase in the release of atrial natriuretic peptide.^8,12^ Together, these responses lead to an approximately 10%–15% reduction in blood plasma volume. Moreover, it is worth noting that these reductions in plasma volume do not result from increased diuresis or natriuresis, but rather likely from reduced interstitial pressures and increased upper body vascular pressures, which together encourage transcapillary fluid movement into the upper body's interstitium.^12,13^ Of note, long-duration spaceflight has also resulted in cardiac remodeling, namely atrophy.^14^ For example, a 2001 study found that left ventricular mass decreased an average of 12% ± 6.9% in four male astronauts after only 10 days in space.^15,16^ Additionally, as a result of decreased plasma volume, the concentration of circulating red blood cells (RBCs) increases, which in turn prompts the body to destroy newly released or nascent red blood cells in another attempt to reestablish homeostatic balance.^8,17^^–^^19^ Upon the conclusion of short-duration space flights of 10–14 days, individuals lost on average 10%–15% of their hematocrit as measured immediately after landing, thereby corresponding to a loss of approximately 1% RBC mass per day.^20^^–^^22^ However, interestingly, Kunz *et al*. report that long-duration spaceflights actually see an increase in the concentrations of both RBCs and hemoglobin, which may suggest that spaceflight anemia will prove less of a potential problem as the body continues to acclimate to microgravity conditions, although the exact mechanism by which it achieves this remains unclear.^20^ Moreover, Garrett-Bakelman *et al*. report that long-duration spaceflight, while also resulting in a decrease in systolic and mean arterial pressure, interestingly results in a 10% increase in carotid diameter, and cardiac output.^4^ Previous studies examining cardiac output changes in spaceflight support the findings of Garret-Backman *et al*., namely, that long-duration spaceflight might actually increase stroke volume and cardiac output by as much as 35% and 41%, respectively, which contradict earlier findings of decreased or unchanged stroke volume and cardiac output during prolonged trips to the International or Russian Space Stations.^23^^–^^26^ As Norsk *et al*. astutely observe, the contradictory data provided by Herault *et al*.^25^ and Hamilton *et al*.^26^ might contradict those provided by Norsk *et al*.^23^ because of the use of differing reference postures on Earth, with the former two studies placing their subjects in supine positions prior to flight, which increases measured cardiac workload by 15%–29%.^10^ Of note, the Twins Study estimated cardiac output of the flight subject via measurements of their diastolic carotid diameter in the supine position both on Earth and in space, while Norsk *et al*. employed a foreign gas rebreathing technique and recorded their measurements in the seated position.^4,23^

## Cardiomyocyte alterations in microgravity

**Figure 1.**
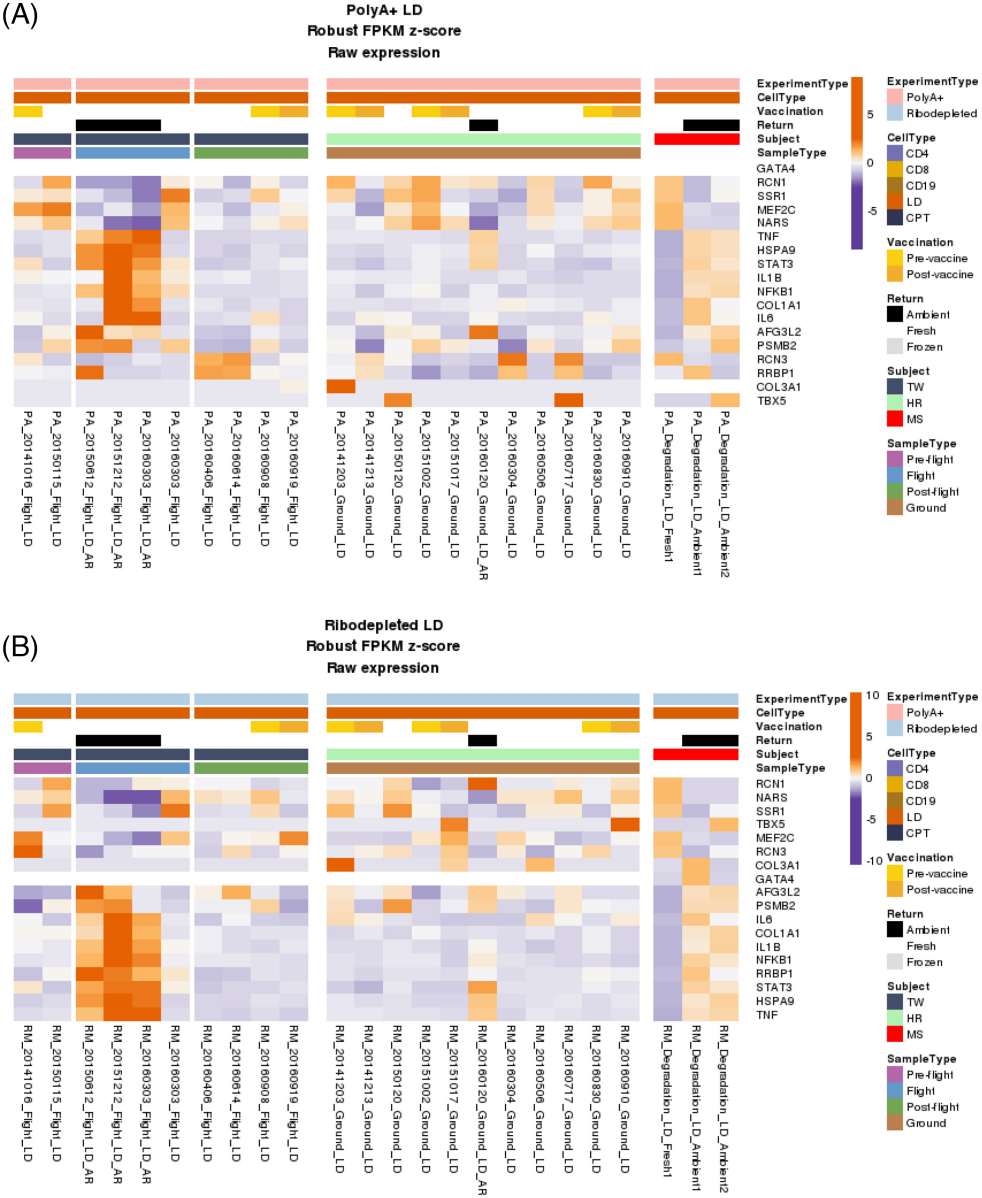
Gene expression profiles. The differentially expressed genes of (A) polyA + lymphocyte depleted and (B) ribodepleted lymphocyte depleted cell types showing upregulated (orange) genes as well as downregulated (purple) genes.

The basic functional unit of contractile cardiac tissue is the cardiomyocyte: a mononuclear, non-proliferative, striated muscle cell with a high density of mitochondria. As such, modulations in cardiomyocyte gene expression resulting from microgravity conditions or radiation exposure are therefore of high importance to NASA in its attempt to embark upon long-term space missions. Figure 1 depicts a summary of differential gene expression profiles of cardiac genes of interest from Garrett-Bakelman *et al*.^4^ As delineated above, microgravity places an enormous stress on the heart, which results in cardiac adaptation. Why and how this occurs on a cellular level, however, remains to be fully elucidated. An *in vitro* study of rat cardiomyocytes by Feger *et al*. asserts that microgravity induces physiological adaptation via alterations in the protein content and function of the mitochondria, ribosomes, and endoplasmic reticulum (ER), ultimately resulting in decreased protein synthesis and turnover, and therefore atrophy.^27^

Cell type, stressor type, stressor duration, and cellular proliferation status each play a role in guiding the type of stress response induced within a cell.^27^ The three types of stress responses at the level of the proteome are the cytoplasmic “heat shock” response, the unfolded protein response (UPR) occurring in the ER, and the UPR occurring in the mitochondria.^27^ Cardiomyocytes exposed to 120 hours of microgravity simulated by placement in a rotating wall bioreactor demonstrated increased numbers and activity of mitochondria, which, in a healthy cardiomyocyte, occupy >30% of cardiac cell volume, while those of both the ribosomes and ER were downregulated.^27^ Specifically, the mitochondrial import protein mortalin, a pro-survival chaperone, and AFG3L2, a protein that assists in degrading defective components of the electron transport chain, were upregulated.^27^ In contrast, in the ribosome, the 40 S components S28, S9-like, and S14-like, the 60 S components L30-like and L12, and ribosomal protein L4 each decreased in abundance.^27^ Additionally, concentrations of asparaginyl-tRNA synthetase, an enzyme involved in rough ER protein synthesis, as well as ribosome binding protein 1, a mediator of the interaction between rough ER and ribosome, were also decreased.^27^ Translocon-associated protein subunit α precursor, reticulocalbin 3, reticulocalbin-1 precursor, and proteasome subunit β type-2, all proteins involved in folding of gene products, were each also diminished upon exposure to microgravity.^27^ In short, it appears as though protein translation via the rough ER decreases in cardiomyocytes exposed to microgravity; cells under duress may be able to shunt their major protein synthesis efforts from the rough ER to the mitochondria to ensure ATP production via mortalin-mediated action.^27^ Of note as well in this study was a significant decrease in cytoskeletal components responsible for mitochondrial localization, namely myosin regulatory light chain and tropomyosin, which is characteristic of muscle atrophy.^27^ In 2019, NASA published the results of the “Twins Study”, a multidimensional comparison of novel genetic and physiologic differences arising between two twins, one of which was earthbound and the other of which completed a 340-day mission at the International Space Station.^4^ Of relevance, the Twins Study flight subject showed increased levels of mitochondrial DNA and RNA in the peripheral blood, especially early in the mission, which may represent those lost during atrophy and apoptosis or ejection of mitochondria from lymphocytes.^4^ Ultimately, the space environment itself may contribute to the cardiac muscle atrophy, as described in the preceding paragraphs, wherein cells in microgravity may maintain mitochondrial function at the expense of rough ER-mediated protein synthesis.^27^

## Arrhythmias during spaceflight

Another of NASA's concerns regarding long-term spaceflight is the potential for cardiac arrhythmias, or changes in the normal sequence of the electrical impulses responsible for coordinated atrial and ventricular contraction. Common arrhythmias include atrial or ventricular fibrillation, or disorganized regional depolarization, bradycardia, or a slower than normal heart rate, tachycardia, or a faster than normal heart rate, premature contractions, and other conduction disorders.^28^ Cardiac arrhythmias cause the heart to pump less effectively, which increases the risk for sudden cardiac death, stroke, cardiovascular disease, and dementia.^29^ Although only anecdotal data exist regarding the occurrence of arrhythmias resulting from spaceflight, they appear to be frequent, albeit transient.

The earliest reports of cardiac arrhythmias occurred during the Apollo era (1961–1972), in which astronaut James Benson Irwin experienced premature ventricular contractions associated with hypokalemia during the Apollo 15 mission, and were reported on several instances throughout the Skylab missions of the subsequent decade (1973–1979).^15^ Upon the conclusion of the MIR era (1986–2001), the Russian Federation reported a total of 75 arrhythmias and 23 conduction disorders to NASA, including an instance of a 14-beat episode of ventricular tachycardia with a maximum rate of 215 bpm. While several causative factors including electrolyte disturbance, alterations in the autonomous nervous system, and changes in the mass of cardiac chambers have been postulated to be the causative agents of that event, the possible triggers remains uncertain.^15^ Increasing evidence has also elucidated conduction problems that are unique to long-term spaceflight. For example, prolongation of the corrected QT interval of the electrocardiogram reading, traditionally associated with an increased risk of Torsades de Pointes, a myocardial repolarization disorder, as well as increased arrhythmia susceptibility, have been observed in longer (4 month), but not short-duration space flights.^30^

## Cosmic radiation and the heart

Of central importance for long-term spaceflight is the hazard of space radiation and cosmic weather to the heart. It is estimated that one-third of ionizing radiation (IR)-induced mortality after the atomic bombings of Japan resulted from cardiovascular disease (CVD) and stroke.^31^ Additionally, ionizing radiotherapy (RT) has been widely known to induce CVD in patients with cancer.^32^ Even in healthy individuals, it has been well established that exposure to high doses of ionizing radiation can induce heart disease, manifesting in the form of accelerated atherosclerosis, myocardial fibrosis, valve abnormalities, conduction deficiencies, and arrhythmia, with cellular injury occurring within minutes of exposure.^33,34^ Moreover, specific effects of radiation have been reported to compromise the integrity of the coronary arteries. For example, exposure to a single physical dose of 0.1–0.2 Gy of radioactive iron ions, the equivalent of a radiation dosage from entrance into the Earth's orbital plane (e.g. 400 km from the surface), has been shown to result in degenerative change in murine coronary arteries, manifest as fibrosis, smooth muscle degeneration, and extracellular deposition, 15 months after exposure to radiation.^35^ Darby *et al*. also estimate a linear increase in major adverse coronary artery events of 7.4% per Gy administered, with no detectable lower or upper threshold.^36^

Quantified, it is predicted that a radiation dose of cumulative of 0.5–1.0 total Sieverts (Sv) will be experienced by an individual traveling to Mars.^8,37^ Thus, a 1000-day exploratory mission to Mars, which would require approximately 400 days in space and 600 days on the surface of Mars, would result in an increased lifetime risk of death from radiation exposure between 1.3% and 13% for a 40-year-old male.^15^ Whole body IR exposure has been shown to increase aortic stiffness and *ex vivo* aortic tension even eight months after a single dose of 1-Gy exposure, with increased aortic lesions, carotid intima thickening, and increases in atherosclerosis observed in apoE deficient mice after exposure to iron radiation at dosages of 2 to 5 Gy.^32,38,39^ Long-duration spaceflight has also been demonstrated to result in carotid intima-media thickening, with levels of collagen α-1(III) chain (COL3A1) and collagen α-1(I) chain (COL1A1) increased in urine inflight, as well as enrichment of collagen-associated gene expression (Fig. 1), both of which may elucidate a mechanism by which the carotid intima and media thicken during space flight and accompanying radiation exposure.^4^ It is also crucial to note that cellular responses to IR vary in a radiation-type-dependent manner, and that cytokines and transcriptions factors, namely NF-κB, have been shown to be modulated specifically by exposure to high-energy heavy ions (HZE) particles.^32,40^

In one *in vivo* study of murine cardiomyocytes, analysis after 7, 14, and 28 days post-IR exposure (15 cGy, 1 GeV/n dose of 56Fe-IR) demonstrated an overall increase in pro-inflammatory, free-radical scavenging, and cardiovascular development and maintenance pathways.^32^ More specifically, modulation was seen in expression of transcription factors such as STAT3, GATA4, and p38 MAPK signaling, with a considerable overlap between neurodegenerative and cardiac muscle disorder-specific transcripts, thereby suggesting a common mechanism by which IR induces both neurodegenerative and cardiac disorders.^32^ Moreover, the upregulation of cardioprotective transcription factors such as TBX5, GATA4, and MEF2C occurred by day 14, well before the onset of any detectible symptoms of cardiac dysfunction. In fact, symptoms first appeared one month after exposure to ^56^Fe-IR, with mice demonstrating both systolic and diastolic dysfunction.^32^ More specifically, levels of STAT3 were lower than that of control at the 14-day time mark, and increased by the 28-day timemark, while levels of GATA4 were consistently upregulated at the 7-, 14-, and 28-day timemarks, with NF-κB showing consistent suppression at the 7-, 14-, and 28-day timemarks.^32^ The authors postulate that such suppression of NF-κB could be a cellular attempt to compensate for the onset of pressure overload-induced cardiac dysfunction.^32^ It is also worth noting that levels of STAT3 phosphorylation were maintained at similar levels to control at day 7 and day 14, but experienced a three- to six-fold increase at the day 28 timemark with respect to control.^32^ p38 phosphorylation was significantly increased at both the 7- and 14-day time marks, but was significantly decreased with respect to control at the 28-day timemark.

These data also indicate a delayed immune response to radiation in cardiomyocytes as well. For example, 14 days after exposure to ^56^Fe-IR, cells exhibited a two-fold increase in macrophage infiltration, with a slight decrease by day 28, which was still maintained at a concentration 35% higher than normal.^32^ However, more research must be done to examine the effect of long-term exposure to low-dose radiation and the subsequent inflammatory response, as prolonged macrophage stimulation is capable of damaging tissue via oxidative stress.

Moreover, the specific type of radiation in space, manifest in the form of protons and, beyond the Van Allen belts (HZE particles) differs from those commonly experienced on Earth, and is postulated to have more biologically deleterious effects than that from Earth's X-rays or γ-rays.^8^ Therefore, given that the nucleus of every cell in the body will be traversed by a high energy proton once every three days *en route* to Mars, it is highly valuable to study the effect of proton radiation on the heart as well.^41^ Boerma *et al*. found that high energy protons, as well as radioactive iron ions, induced migration of CD68+ monocytes and macrophages into cardiac tissue, increased DNA oxidation, myocardial fibrosis, and decreased cardiac function in murine models in a radiation-type specific manner.^33^ Additionally, exposure to radioactive silicone ions resulted in prolonged apoptosis and increased expression of pro-inflammatory cytokines such as interleukin (IL)-1β, IL-6, and tumor necrosis factor-α in cardiac tissue, while exposure to low doses of high-linear energy transfer radiation has been shown to modulate DNA methylation, thereby bringing epigenetic control to the potential forefront of the cardiovascular response to radiation.^33^ Notably some of these same pro-inflammatory markers (e.g. IL-6) were also observed in the Twins Study.^4^

## Re-acclimation and orthostatic intolerance

Finally, upon returning to Earth, orthostatic intolerance is frequently encountered, with presyncopal symptoms, defined as light-headedness, dizziness, or nausea, reported in 28%–65% of astronauts performing stand or tilt tests upon return, which may also prove problematic for successful activities on the surface of Mars—even at 36% of the gravity.^6,42^ More simply, orthostatic intolerance could pose a life-threatening condition in the event of an emergency evacuation upon re-exposure to a gravitational force as well. On Earth, orthostatic intolerance has many causes, including decreases in stroke volume, plasma volume and constriction of restriction vessels.^6^ Orthostatic events experienced by both short-duration and long-duration astronauts range from transient tachycardia to orthostatic hypotension (systolic blood pressure below 70 mmHg while standing) and presyncope.^43^ It is worth noting, therefore, that orthostatic intolerance in astronauts exposed to long-duration spaceflight (129–190 days) is considerably increased with respect to their short duration controls, with previous studies reporting symptoms of orthostatic intolerance in 20%–30% of astronauts returning from short-duration flights, and 83% in those returning from long-duration flights.^43,44^ Despite NASA providing astronauts with a fluid-loading protocol prior to re-entry, terrestrial plasma volume has still been shown to be reduced by 7%–20% compared to preflight.^43^ Also, a recent study on venous outflow^45^ has shown that stagnant or reverse flow in the internal jugular vein (IJV) is frequent (6/11 crew members, or 55%) by mid-flight (day 50) for short-duration missions. Also, one crew member had an occlusive IJV thrombus, and a potential partial IJV thrombus was identified in another crew member retrospectively, indicating blood flow issues that warrant future monitoring in upcoming missions.

Upon landing after the missions, there are still more considerations. Astronauts returning from long-duration spaceflights showed greater decreases in stroke volume and were completely unable to maintain upright arterial pressure upon return from long-duration flights, despite decreases in plasma volume being comparable between astronauts participating in short- and long-duration flights.^44^ Additionally, astronauts returning from long-duration spaceflights demonstrated an attenuated adrenergic response, showing decreased levels of norepinephrine upon return attempts to stand; conversely, these individuals demonstrated five times higher levels of epinephrine than short-duration astronauts, which suggests an acute stress response not present during the re-acclimation of short-duration astronauts.^44^ All of these factors would benefit from improved imaging and molecular diagnostics during the mission, to better assess the risk for astronauts.

## Conclusions

While the effects of spaceflight on the heart have been studied since the first manned missions into Earth's orbit, there is still a great deal of information that remains controversial, contradictory, or simply unknown. Further investigation of the effects specific to long-term spaceflight, namely, on epigenetic and genetic alterations induced by radiation, stress, or microgravity, for example, is essential to most thoroughly inform NASA and other groups of methods to develop preventative countermeasures against potential harms exploratory travel might inflict on an astronaut bound for any planet beyond our own. Moreover, the implications for clonal hematopoiesis^46^ and heart function are not yet known, and could be significant for planning long-term missions. Finally, while many of these metrics require samples to be returned from the ISS for analysis, new methods that can sense a range of biomolecules have been shown to work in microgravity^47^ and on the ISS^48,49^ and could also help decease the time interval between measurements for some of these key metrics.
